# Sick and depressed? The causal impact of a diabetes diagnosis on depression

**DOI:** 10.1186/s13561-023-00451-w

**Published:** 2023-07-03

**Authors:** Alessio Gaggero, Joan Gil, Dolores Jiménez-Rubio, Eugenio Zucchelli

**Affiliations:** 1 Department of Applied Economics, University of Granada, Granada, Spain; 2 Department of Economics and BEAT, Universitat de Barcelona, Diagonal Ave. 696, 08034 Barcelona, Spain; 3 Department of Applied Economics, University of Granada, Granada, Spain; 4 Madrid Institute for Advanced Study (MIAS) and Department of Economic Analysis, Universidad Autónoma de Madrid (UAM), Madrid, Spain; 5 Lancaster University, Lancaster, UK; 6 IZA, Bonn, Germany

**Keywords:** Diabetes, Depression, Fuzzy regression discontinuity design, Administrative longitudinal data, Lifestyle changes

## Abstract

**Background:**

There is sparse evidence on the impact of health information on mental health as well as on the mechanisms governing this relationship. We estimate the causal impact of health information on mental health via the effect of a diabetes diagnosis on depression.

**Methods:**

We employ a fuzzy regression discontinuity design (RDD) exploiting the exogenous cut-off value of a biomarker used to diagnose type-2 diabetes (glycated haemoglobin, HbA1c) and information on psycometrically validated measures of diagnosed clinical depression drawn from rich administrative longitudinal individual-level data from a large municipality in Spain. This approach allows estimating the causal impact of a type-2 diabetes diagnosis on clinica ldepression.

**Results:**

We find that overall a type-2 diabetes diagnosis increases the probability of becoming depressed, however this effect appears to be driven mostly by women, and in particular those who are relatively younger and obese. Results also appear to differ by changes in lifestyle induced by the diabetes diagnosis: while women who did not lose weight are more likely to develop depression, men who did lose weight present a reduced probability of being depressed. Results are robust to alternative parametric and non-parametric specifications and placebo tests.

**Conclusions:**

The study provides novel empirical evidence on the causal impact of health information on mental health, shedding light on gender-based differences in such effects and potential mechanisms through changes in lifestyle behaviours.

**Supplementary Information:**

The online version contains supplementary material available at 10.1186/s13561-023-00451-w.

## Background

An increasing body of evidence suggests the relevance of health information in influencing key health-behaviours. For instance, the medical literature finds that the information provided by portable devices [1, 2] or the diagnosis of specific types of cancer [3, 4] might trigger behavioural changes and ultimately affect health outcomes. More recently, the economics literature has started exploring the role of health information by focusing on the impact of the diagnosis of chronic conditions, including hypertension and diabetes [5–9]. While these recent economics studies often employ causal inference methods and are thus capable of identifying causal effects, they mostly focus on changes in health-behaviours while ignoring other relevant health outcomes such as mental health.

Mental health and depression have been consistently found to be linked with health-behaviours. A series of studies show that individuals with healthier patterns of lifestyle behaviours present lower levels of psychological distress [10–16]. In addition, the relationship between chronic conditions and mental health is well-documented with several papers suggesting strong correlations between major chronic conditions such as diabetes and mental health. More specifically, a large body of evidence finds strong and sizeable correlations between a diabetes diagnosis and several mental health outcomes such as clinical depression, consumption of antidepressants, and measures of quality of life and social interactions [17–22]. Equally, another stream of studies shows significant associations between mental ill-health and the risk of developing T2DM [17, 22–24]. However, the existing literature does not appear to have comprehensively investigated the role of health information in causally influencing mental health.

The main objective of this paper is to identify the causal impact of health information on mental health via the impact of a diagnosis of type-2 diabetes (T2DM) on clinical depression using a regression discontinuity design (RDD). More specifically, we exploit the discontinuity offered by the exogenous cut-off of a biomarker commonly used for the diagnosis of T2DM (i.e. glycated haemoglobin, HbA1c) to estimate the impact of a T2DM diagnosis on diagnosed clinical depression using rich longitudinal administrative data from Spain.

This paper offers several contributions to the literature. First, we provide novel causal evidence of the impact of health information on depression, one of the most widespread mental disorders affecting around 280 million individuals globally [25]. While the growing literature on the role of health information has mainly focused on its effect on health-behaviours especially among individuals with chronic conditions, to the best of our knowledge no previous studies have attempted to identify the causal impact of health information on diagnosed clinical depression. Second, our analysis suggests that the effect of a type-2 diabetes diagnosis on depression might vary by gender, age and BMI level as well as by the behavioural changes induced by it specifically weight loss. Hence, this contributes directly to the literature on the relevance of health information and the mechanisms through which it might affect health outcomes. From a policy perspective this might be also potentially useful as it highlights that the provision of health information (in the form of a diabetes diagnosis) could positively affect both lifestyle hehaviours (weight losses in particular) and mental health. Third, our empirical analysis also contributes to the large strand of the literature concerned with the determinants of mental health among individuals with major chronic conditions, including obesity and type-2 diabetes. This is also likely to be relevant policy-wise as type-2 diabetes is currently affecting 537 million individuals, and its burden of disease is projected to increase in both developing and developed countries [26]. Finally, differently from the majority of previous studies on the impact of a T2DM diagnosis employing a sharp RDD, the *fuzzy* RDD approach used here allows accounting for the possibility that a T2DM diagnosis may not be exclusively based on the cut-off of a single biomarker, but on a wider set of information including family history and the presence of comorbidities.

More generally, estimating the causal impact of a T2DM diagnosis on depression might be relevant from an economic perspective as these are both highly prevalent conditions with significant impacts on the quality of life and productivity of individuals. Indeed, both T2DM and mental ill-health greatly affect an individual’s ability to work leading to reduced labour force participation and increased absenteeism [27–30]. Hence, establishing and quantifying a causal relationship between these two widespread conditions, might provide valuable information to develop more targeted interventions. These should be aimed at reducing the combined economic burden of T2DM and depression, including screening and early detection programmes for patients with diabetes and appropriate treatment strategies more explicitly accounting for the potential risk of clinical depression.

### Previous literature

Several recent studies in the field of medicine have explored the effects of health information either via the diagnosis of specific types of cancers or by portable devices on clinical outcomes as well as risky health-behaviours [1–4]. However, these findings are mostly based on standard statistical associations and are either mixed or observed only among specific sub-groups of individuals.

The economics literature has also started exploring the role of health information in influencing health and health-behaviours. This is highly relevant as standard economic models assume that individuals have complete knowledge about their health, and they can perfectly and rationally process it when making health investment decisions [31, 32]. However, this assumption has been recently re-assessed by empirical and experimental studies [33–35]. Early economic studies focus on the effects of public health information campaigns [36–39] or nutritional labels [40, 41] while more recent contributions attempt to identify the causal impact of health information via the diagnosis of chronic conditions, including T2DM, on lifestyle behaviours [5–9] and cardiovascular risk factors [42]. The latter studies tend to increasingly find significant causal impacts of a T2DM diagnosis mostly on weight loss or fat intake.

Moreover, the dual relationship between diabetes and mental health has been investigated, mainly in the medical literature. Several of these studies have shown a significant association between the diagnosis of T2DM and the deterioration of mental health [17–20] with negative effects on quality of life, social contacts [20, 22], and increases in the consumption of antidepressants [18]. In addition, a consistent finding in the medical literature is that major depressive disorders increase the risk of developing T2DM and subsequent complications [17, 23, 24].

Importantly, most medical evidence tends to rely on self-reported information of key variables of interest and only identifies standard statistical correlations, while overlooking potential endogeneity issues. Gaggero [7] appears to be among the very few economics studies attempting to identify the effect of a diabetes diagnosis on a measure of mental health. However, he only employs self-reported information on mental health together with a less reliable biomarker, i.e. Fasting Plasma Glucose, [43] to detect diabetes on a sample of older individuals in England, finding no statistically significant effects of a T2DM diagnosis. As a result, the literature has not yet established whether health information may have a *causal* impact on developing a diagnosed mental health condition and, more specifically, whether a T2DM diagnosis might causally affect clinical depression.

## Methods

### Data

We employ administrative data of patients followed over a seven-year period (2004–2010) drawn from six GP practices and two hospitals located in the city of Badalona (nort-east of Barcelona), Spain, an EU country with a universal health care system free at the point of delivery [44]. The initial sample includes patients aged 16 + who had at least one contact with those hospitals and centres between 1 January 2004 and 31 December 2010.^1^

The dataset contains detailed information about patients’ clinical measurements of height and weight and any diagnosed health condition, including clinical or major depression.^2^ More specifically, clinical depression is identified by a binary variable taking value 1 if the patient is diagnosed with clinical/major depression, corresponding to the code/registry P76 of the International Classification of Primary Care, second edition (ICPC-2), 0 otherwise. The information used by physicians to diagnose depression is based on a series of psychometrically validated measures of clinical depression, including the Golberg Anxiety and Depression Scale (GADS), the Hamilton Rating Scale for Depression (HRSD) as well as the Geriatric Depression Rating Scale (GDRS) [45–48]. All three measures are collected by physicians through interviews with patients on the basis of a series of items identifying several symptoms of depression experienced by the patients during the previous week (18 in the GADS; 17 in the HRSD; 15 items in the short form of the GDRS index or 35 in the longer version, respectively). While the GADS and the HRSD can be used to detect depression in the general population, the GDRS is specifically designed to diagnose depression among older patients. The latter measure might be particularly useful in this case given the average age of individuals in our sample. The standard recommended thresholds to diagnose clinical depression for each validated measure were used.^3^

### Other key variables and descriptive statistics

Our medical records also include data on glycated haemoglobin (henceforth, HbA1c), a biomarker providing a measure of a patient’s average blood sugar level in the previous 8–12 weeks that is commonly used to diagnose T2DM [52]. In our setting, physicians follow standard national and international medical guidelines for patients with T2DM and use the threshold value of HbA1c ≥ 6.5 percent to diagnose T2DM [53].^4^ This test is administered as part of routine health checks to all individuals presenting relevant risk factors or symptoms of hyperglycaemia (high blood sugar levels).

HbA1c measurements are endorsed by the International Expert Committee (IEC) and the American Diabetes Association (ADA) as they are more reliable if compared to other measures of blood sugar such as the Fasting Plasma Glucose (FPG).^5^ The latter appears to have a substantially shorter time validity; to be sensitive to short-term lifestyle changes and stress; and tend to systematically underestimate the prevalence of diabetes [43, 52, 58].^6^ Relevant to this study, upon a T2DM diagnosis, patients of the Spanish health care system are normally recommended to follow a non-pharmacological treatment consisting of educational training sessions for diabetes self-management aimed at improving their lifestyle though dietary changes and regular exercise.

In addition, the dataset includes a rich set of demographic and socioeconomic characteristics such as age; gender; employment status (active/retired); marital status (married/cohabiting vs living alone); immigration status (EU vs non-EU), that we use as control variables. For the purpose of our analysis, we include in our estimating sample individuals with at least one biomarker measurement per year. This effectively includes any individual either diagnosed with diabetes; at risk of diabetes (including pre-diabetics, i.e. patients with a HbA1c value between 5.7–6.4 percent); or any other individual with relevant risk factors or symptoms that may lead to high blood sugar levels. This leads to a sample of 13,971 individuals (39,994 obs.).^7^

Table 1 reports the summary statistics of the main variables of interest. The Table shows that around 18 percent of the patients are diagnosed with clinical depression.^8^ We next report statistics on individuals diagnosed with T2DM via the corresponding ICPC-2 code informed by the HbA1c values. The average HbA1c for the patients in our sample is around 6.6 percent and on average patients have been diagnosed for a little over 3 years (see the variable labelled “onset”). With respect to other demographic variables, the average age of the sample is around 65, and the sample is almost evenly split by gender. Furthermore, 87, 27 and 2 percent of the sample are, respectively, living with a partner; active in the labour market; and were born outside the EU. Finally, the Table also reports that 59 and 53 percent of the patients are also diagnosed with hypertension and dyslipidaemia (the presence of high amounts of lipids, including cholesterol, in blood), respectively; while 4, 7 and 5 percent of the patients are affected by asthma, neoplasms/cancers and chronic obstructive pulmonary disease (COPD) respectively. Tables S1-S3 in the Additional file 1 show differences in individuals’ characteristics by treatment status, gender and bodyweight change.

**Table 1:** Summary statistics

	Mean	S.D	Min	Max	Obs
**Outcome Variable:**					
Depression [0,1]	0.18	0.38	0	1	39,688
**T2DM Variables:**					
T2DM Diagnosis [0,1]	0.67	0.47	0	1	39,688
Onset of T2DM	3.13	3.72	0	39	34,741
HbA1C (%)	6.60	1.43	0	20	39,994
**Demographics:**					
Years of Age	65.10	12.62	16	106	39,688
Female [0,1]	0.52	0.50	0	1	39,688
Not Living Alone [0,1]	0.87	0.33	0	1	39,688
Active [0,1]	0.27	0.44	0	1	39,594
Immigrant [0,1 l	0.02	0.13	0	1	39,688
**Other Conditions:**					
Hypertension [0,1]	0.59	0.49	0	1	39,688
Dyslipedimia [0,1]	0.53	0.50	0	1	39,688
Asthma	0.04	0.21	0	1	39,688
Neoplasms-cancer [0,1]	0.07	0.25	0	1	39,688
COPD	0.05	0.23	0	1	39,688
Observations	39,994				

The Table reports summary statistics of the main variables of interest

### Empirical approach

We employ a *fuzzy* regression discontinuity design (RDD) exploiting the discontinuity offered by the exogenous cut-off value of 6.5 of the biomarker (HbA1c) used to diagnose T2DM. We choose to follow a fuzzy design as physicians may not base their diagnosis solely on HbA1c values. For instance, they could potentially look at further patients’ characteristics as well as family history around T2DM or whether individuals may suffer from other metabolic conditions, such as hypertension or dyslipidaemia. For instance, it might be the case that some physicians may diagnose with T2DM individuals with several metabolic conditions and a value of HbA1c just below 6.5 percent. Our fuzzy RDD approach would allow accounting for such cases.^9^

A fuzzy RDD estimation is akin to a two-stage least squares instrumental variable (2SLS-IV) specification [60]. Accordingly, the first stage equation can be represented as follows:

1 $${D}_{i,t}=\mu +\rho {Above}_{i,t}+\tau {Above}_{i,t}\left({HbA1c}_{i,t}-6.5\right)+\delta \left({HbA1c}_{i,t}-6.5\right)+ {{\varvec{X}}}_{{\varvec{i}},{\varvec{t}}}^{\boldsymbol{^{\prime}}}\Omega +{\epsilon }_{i,t}$$

where ${D}_{i,t}$ is a dummy indicator for whether individual $i$ was diagnosed with T2DM at time $t$ (i.e., year).$Abov{e}_{it}$ is a binary variable that takes the value of 1 when the HbA1c value of an individual is larger than or equal to the predetermined cut-off value of 6.5, defining treatment assignment, and acts as the instrument for the T2DM diagnosis. The covariate $\left({HbA1c}_{i,t}-6.5\right)$ is the centred or normalised HbA1c.^10^ There are two main alternative ways for selecting the functional form to estimate the magnitude of the discontinuity in the outcome of interest at the cut-off point within a RDD setting: the parametric approach and the non-parametric approach. While the parametric approach focuses on the optimal functional form to fit the full data, the nonparametric approach focuses on an arbitrarily small neighbourhood sample around the cut-off. To make full use of our estimating sample, and following Gelman and Imbens^11^ [61], our main specification considers a linear polynomial of the running variable. However, we examine the robustness of our results to higher order polynomials and non-parametric estimations based on a local randomization approach [62]. All estimations control for a vector of covariates ${{\varvec{X}}}_{{\varvec{i}},{\varvec{t}}}^{\boldsymbol{^{\prime}}}$ including sociodemographic characteristics (age, gender, employment, marital and immigrant status). We also control for several pre-diagnosed conditions, including hypertension, dyslipidaemia, asthma, neoplasms/cancers, and COPD as well as time elapsed from the diagnosis. Additionally, our econometric specifications include time-, health area- and GP fixed-effects (FE). This allows controlling for any systematic (time-invariant) differences across physicians that might affect the diagnoses of T2DM and major depression.

The second stage equation can be written as:

2 $${Y}_{i,t+1}=\alpha +\beta {D}_{i,t}+\theta \left({HbA1c}_{i,t}-6.5\right)+ {{\varvec{X}}}_{{\varvec{i}},{\varvec{t}}}^{\boldsymbol{^{\prime}}}\gamma +{\varepsilon }_{i,t}$$

${Y}_{i,t+1}$ denotes the outcome of interest and measures whether individual $i$ is diagnosed with clinical depression at time $t+1$, that is *after* the T2DM diagnosis, conditional on not having been diagnosed at time t. As above, it is assumed a linear function (although other polynomials are further examined) of the normalised running variable, and this is allowed to vary around the cut-off. ${{\varvec{X}}}_{{\varvec{i}}{\varvec{t}}}^{\boldsymbol{^{\prime}}}$ is the same vector of covariates discussed above. Finally, ${\varepsilon }_{i,t}$ is a random error term. We cluster standard errors on the running variable based on the recommendation of Lee and Card [63].^12^ The main term of interest is $\beta$ as it measures the change in the probability of being diagnosed with depression following a T2DM diagnosis. This coefficient captures the local average treatment effect (LATE) of a diabetes diagnosis among the group of compliers around the cut-off [65]. To make full use of the sample size, we estimate Eqs. (1) and (2) parametrically. However, we further explore the robustness of our results using the non-parametric approach mentioned above as part of our sensitivity analysis.

### RDD validity

In order to test whether the average outcome of those just below the cut-off can be used as the counterfactual for those above the cut-off, we provide two indirect tests suggesting the overall credibility of our RDD application [66].

**Fig. 1:**
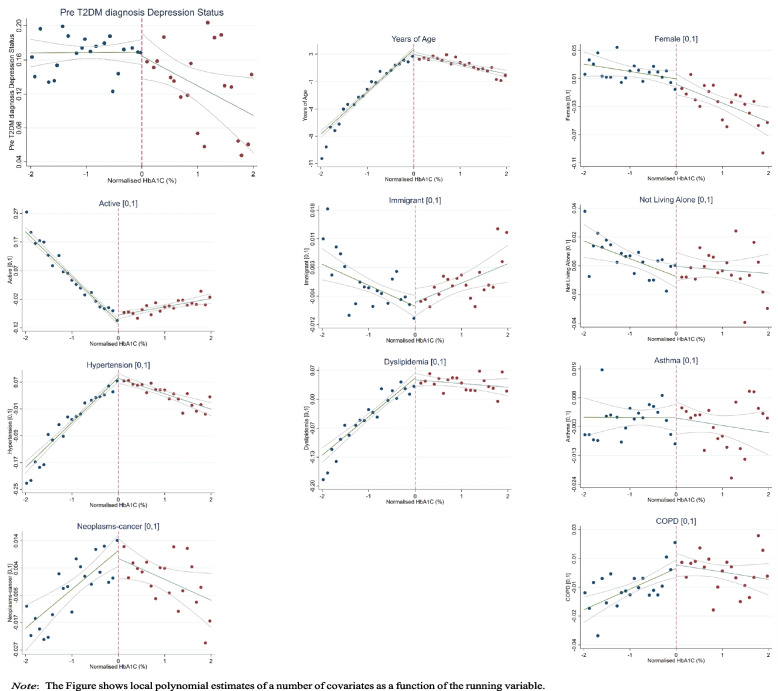
Continuity Test

First, we examine whether there are any significant differences in pre-determined characteristics at the cut-off point. Ideally, we should find null effects of the diagnosis on these characteristics. The results of this exercise are presented in Fig. 1. Each graph presents the local polynomial smoothing (LPS) for pre-diagnosis major depression and covariates as a function of the HbA1c. This confirms the validity of our design by revealing non-significant jumps at the cut-off for any of these variables.

**Fig. 2:**
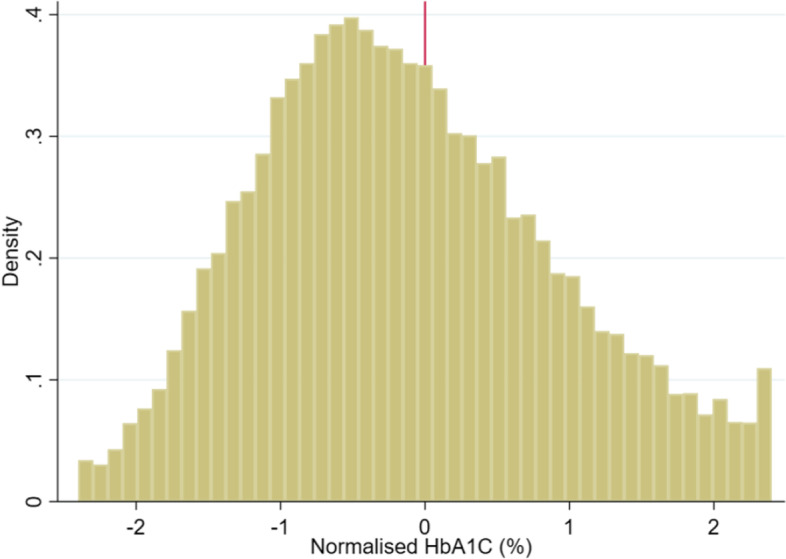
Density of the Running variable. Note: The Figure shows evidence of no manipulation of the running variable. Bin size = 0.1. The bin size has been selected by means of the McCrary test Stata routine, i.e. DCdensity

Second, for the RDD to be valid it is also critical that individuals would not be capable of manipulating their diabetes diagnosis [67]. In our case, it seems highly unlikely that patients could manipulate their HbA1c scores, as this measure is based on a blood test administered by physicians and refers to the average glucose concentration over the previous 8–12 weeks. Yet, in order to rule this out, in Fig. 2 we show the distribution of the density function of the normalised HbA1c around the cut-off, suggesting the absence of any discontinuity, as expected.^13^

## Results

### Main results

**Fig. 3:**
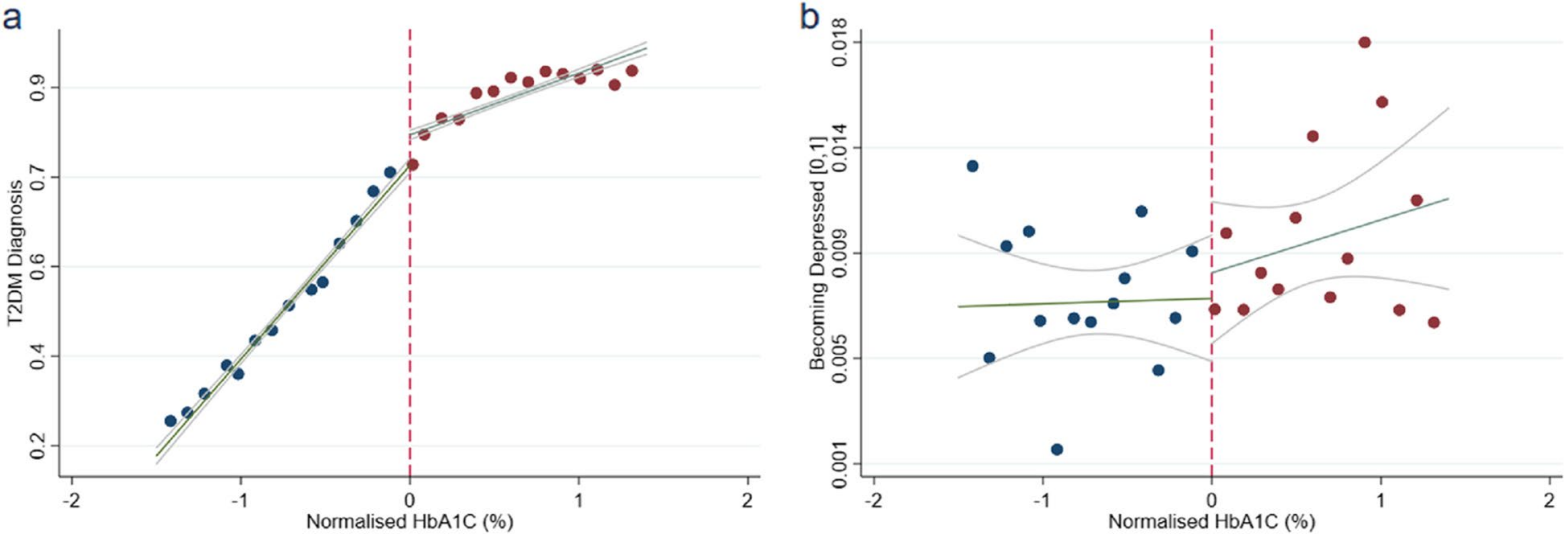
RD Graphical Evidence. Note: **A** shows local polynomial estimates of the probability of being diagnosed with T2DM as a function of the (normalised) HbA1c, our rst stage. Similarly, **b** shows local polynomial estimates of the probability of being diagnosed with depression as a function of the (normalised) HbA1c

Figure 3a-b examine the impact of having a HbA1c level above 6.5 percent. Specifically, Fig. 3a shows a sizeable discontinuity in the probability of being diagnosed with T2DM around the HbA1c cut-off as for our first stage. Similarly, the plot in Fig. 3b implies that patients with normalised HbA1c just above the cut-off are more likely to be diagnosed with depression than their counterparts. We next test the relevance of these findings in a regression framework while controlling for potential confounding factors.

Table 2 reports the RDD estimates of a T2DM diagnosis, as outlined in Eq. (1). Specifically, column (1) includes the basic estimates with no covariates; column (2) adds a set of socioeconomic characteristics; column (3) further accounts for dummies indicating the presence of pre-existing medical conditions; column (4) includes time and area FE; and column (5) finally adds GP FE. Estimates appear statistically significant across all specifications, although as expected, the inclusion of pre-existing conditions appear to recude the size of the effects. According to the most comprehensive specification in column (5), the estimated coefficient implies that patients with an HbA1c above the 6.5 percent cut-off are around 9 percentage points more likely to be diagnosed with T2DM than their counterparts.

**Table 2:** RDD estimates of a T2DM diagnosis

	(1)	(2)	(3)	(4)	(5)
**Above [0,1]**	0.196*** (0.032)	0.193*** (0.031)	0.110*** (0.025)	0.094*** (0.025)	0.088*** (0.025)
Above * Normalised HbA1c	-0.236*** (0.025)	-0.213*** (0.024)	-0.162*** (0.017)	-0.190*** (0.019)	-0.187*** (0.019)
**Running Variable:**					
Normalised HbA1c	0.247*** (0.025)	0.231*** (0.024)	0.163*** (0.017)	0.188*** (0.019)	0.185*** (0.018)
**Attributes:**					
Years of Age		0.001* (0.000)	-0.004*** (0.000)	-0.001*** (0.000)	-0.001*** (0.000)
Female [0,1]		-0.024*** (0.005)	-0.043*** (0.005)	-0.044*** (0.004)	-0.044*** (0.004)
Not Living Alone [0,1]		0.004 (0.005)	-0.021*** (0.005)	-0.016** (0.005)	-0.018*** (0.005)
Active [0,1]		-0.106*** (0.009)	-0.075*** (0.007)	-0.011* (0.005)	-0.012* (0.005)
Immigrant [0,1 l		-0.148*** (0.020)	-0.067*** (0.018)	-0.029 (0.015)	-0.041** (0.015)
Onset of T2DM			0.066*** (0.006)	0.075*** (0.005)	0.075*** (0.005)
**Pre-existing Conditions:**					
Hypertension [0,1]			0.084*** (0.005)	0.056*** (0.004)	0.054*** (0.004)
Dyslipedimia [0,1]			0.054*** (0.003)	0.037*** (0.004)	0.038*** (0.004)
Asthma			0.004 (0.007)	0.008 (0.007)	0.011 (0.007)
Neoplasies-cancer			0.020** (0.007)	0.019** (0.006)	0.017** (0.006)
COPD			-0.005 (0.007)	-0.016** (0.006)	-0.019** (0.007)
**Time FE**				✓	✓
**Area FE**				✓	✓
**GP FE**					✓
Observations	39,688	39,594	34,359	34,356	34,319

The Table reports RDD estimates of a T2DM diagnosis. Although not shown in the Table, estimates are conditional on time, area, and GP fixed effects. Robust standard errors are clustered on the running variable. * *p* < 0.05, ** *p* < 0.01, *** *p* < 0.001

Table 3 reports fuzzy RDD estimates of a T2DM diagnosis of being diagnosed with major or clinical depression. Similarly to Table 2, in columns (1)-(5) we report findings of different specifications including an incremental number of covariates. Here, we observe positive and statistically significant effect of a T2DM diagnosis on clinical depression. In particular, according to our preferred model (column 5), receiving a T2DM diagnosis raises by 1.6 percent the probability of being diagnosed with depression.

**Table 3:** Fuzzy RDD estimates of the impact of a T2DM diagnosis on depression

	(1)	(2)	(3)	(4)	(5)
**T2DM Diagnosis [0,1]**	0.006* (0.003)	0.011 (0.006)	0.011 (0.006)	0.016** (0.005)	0.016** (0.005)
**Running Variable:**					
Normalised HbA1c	0.000 (0.001)	0.001 (0.001)	0.001 (0.001)	0.000 (0.001)	0.000 (0.001)
**Attributes:**					
Years of Age		-0.000 (0.000)	-0.000 (0.000)	0.000 (0.000)	0.000 (0.000)
Female [0,1]		0.006*** (0.001)	0.006*** (0.001)	0.006*** (0.001)	0.006*** (0.001)
Not Living Alone [0,1]		0.002 (0.002)	0.002 (0.002)	0.002 (0.002)	0.002 (0.002)
Active [0,1]		-0.004* (0.002)	-0.004* (0.002)	-0.001 (0.002)	-0.002 (0.002)
Immigrant [0,1 l		-0.006** (0.002)	-0.006** (0.002)	-0.004 (0.002)	-0.004 (0.002)
Onset of T2DM		-0.001** (0.000)	-0.001** (0.000)	-0.001** (0.000)	-0.001** (0.000)
**Pre-existing Conditions:**					
Hypertension [0,1]			-0.000 (0.001)	-0.002 (0.001)	-0.002 (0.001)
Dyslipedimia [0,1]			-0.001 (0.001)	-0.002 (0.001)	-0.002 (0.001)
Asthma			-0.001 (0.003)	-0.001 (0.003)	-0.001 (0.003)
Neoplasies-cancer			-0.003 (0.002)	-0.003 (0.002)	-0.004* (0.002)
COPD			0.000 (0.002)	0.001 (0.002)	0.001 (0.002)
**Time FE**				✓	✓
**Area FE**				✓	✓
**GP FE**					✓
Observations	39,688	34,359	34,359	34,356	34,319

The Table reports RDD estimates of the impact of a T2DM diagnosis on depression. Although not shown in the Table, estimates are conditional on a set of covariates, time, areas and GP fixed effects. Robust standard errors are clustered on the running variable. * *p* < 0.05, ** *p* < 0.01, *** *p* < 0.001

### Heterogeneity and potential mechanisms

Table 4 presents the results of the heterogeneity analysis splitting the data by gender aimed at discerning some potential mediating mechanisms. Panel A shows results for the full sample, whereas Panels B to H present the impacts induced by the T2DM diagnosis stratifying the sample by whether individuals lose weight following the diagnosis, as this is often one of the main lifestyle changes recommended by physicians according to the recent literature [6, 8, 69], by BMI categories (“healthy weight”: 18 <  = BMI < 25; “overweight”: 25 <  = BMI < 30; “obesity”: BMI >  = 30) and also by age (> 60 years and <  = 60 years-old). Columns (1)-(3) report RDD estimates for both genders, and for males and females, respectively.

**Table 4:** Fuzzy RDD estimates of the impact of a T2DM diagnosis on depression—heterogeneity analysis

	(1)	(2)	(3)
	All	Men	Women
**Panel A: Full-Sample:**			
**T2DM Diagnosis [0,1]**	0.016** (0.005)	-0.001 (0.006)	0.032*** (0.008)
Observations	34,319	16,483	17,836
Proportion of Treated	0.67	0.69	0.66
**Panel B: Weight Loss:**			
**T2DM Diagnosis [0,1]**	-0.032 (0.022)	-0.083* (0.037)	0.001 (0.025)
Observations	6990	3111	3879
Proportion of Treated	0.84	0.85	0.83
**Panel C: No Weight Loss:**			
**T2DM Diagnosis [0,1]**	0.032* (0.015)	0.015 (0.016)	0.058* (0.025)
Observations	6862	3242	3620
Proportion of Treated	0.84	0.85	0.83
**Panel D: Healthy Weight:**			
**T2DM Diagnosis [0,1]**	-0.002 (0.014)	-0.010 (0.013)	0.018 (0.026)
Observations	2690	1347	1343
Proportion of Treated	0.69	0.74	0.64
**Panel E: Overweight:**			
**T2DM Diagnosis [0,1]**	0.006 (0.008)	-0.002 (0.011)	0.014 (0.016)
Observations	10,053	5619	4434
Proportion of Treated	0.73	0.74	0.72
**Panel F: Obese:**			
**T2DM Diagnosis [0,1]**	0.024*** (0.007)	0.003 (0.008)	0.041*** (0.010)
Observations	21,539	9502	12,037
Proportion of Treated	0.65	0.66	0.64
**Panel G: Age ≥ 60:**			
**T2DM Diagnosis [0,1]**	0.013* (0.006)	-0.003 (0.008)	0.025** (0.008)
Observations	23,161	10,612	12,549
Proportion of Treated	0.72	0.73	0.71
**Panel H: Age < 60:**			
**T2DM Diagnosis [0,1]**	0.027** (0.009)	0.003 (0.011)	0.055*** (0.015)
Observations	11,158	5871	5287
Proportion of Treated	0.57	0.62	0.51

The Table reports RDD estimates of the impact of a T2DM diagnosis on depression. Although not shown in the Table, estimates are conditional on a set of covariates, time, areas and GP fixed effects. Robust standard errors are clustered on the running variable. * *p* < 0.05, ** *p* < 0.01, *** *p* < 0.001

The estimates in Panel A clearly suggest a gender pattern so that the increase in the probability of developing clinical depression appears to be driven by women: women present an increase of around 3.2 percentage points in the probability of being diagnosed with clinical depression following a diabetes diagnosis, while the corresponding estimate for men is not statistically significant. This appears to be in line with previous findings indicating that male patients with diabetes present higher levels of subjective well-being [70, 71].

We also explore whether losing weight after a T2DM diagnosis might also play a role in explaining the effects on clinical depression. Panels B and C in Table 4 present results for individuals who did versus who did not lose weight, respectively. Importantly, size and statistical significance of these estimates suggest that the observed increase in depression is mostly concentrated among female patients who did not lose weight following a diabetes diagnosis (5.8 percentage points). Yet, men who lost weight after the T2DM diagnosis, are less likely to be diagnosed with major depression (8.3 percentage points). Overall, this appears to suggest the presence of a potentially relevant “protective” effect with respect to the probability of being diagnosed with clinical depression but only among male individuals.^14^

Panels D to F further investigate whether the impact of a T2DM diagnosis on depression might vary by BMI levels. Our estimates show that the increase in depression following a T2DM diagnosis appears to be mainly driven by women in the highest BMI category (4.1 percentage points). Finally, Panels G and H present estimates by two broad age groups (younger and older than 60 years-old) to explore whether age may affect the relationship between a T2DM diagnosis and mental health. Here our findings indicate that the positive and statistically significant impact of a T2DM diagnosis on clinical depression is stronger among the relatively younger age group and also appears to be driven by women. More specifically, women younger than 60 years-old present an increase of 5.5 percentage points in the probability of being diagnosed with clinical depression, while this is lower (2.5 percentage points) among older women.^15^

### Sensitivity analysis

In order to check the robustness of our main results, we also present a series of sensitivity tests. Table S4 shows RDD estimates based on a placebo test consisting of alternative cut-off values of the biomarker. This test should not produce a statistically significant effect for our outcome of interest (clinical depression) at values below the 6.5 percent of the biomarker. Indeed, our placebo test using 5.5, 5 and 4.5 percent cut-off values confirm that the impact on major depression is not statistically different from zero. At the 6 percent value of the biomarker we find statistically significant effects, although smaller in magnitude. Yet, this is expected as prediabetic patients (HbA1c ranging from 5.7–6.4 percent) are normally recommended similar non-pharmacological treatments (dietary changes and regular exercise) [53]. At the 7 percent theshold we find slightly larger effects of a T2DM diagnosis on mental health, and this is also in line with the lifestyle changes recommended by doctors to patients with uncontrolled diabetes.

Table S5 further shows the robustness of our results by estimating the fuzzy RDD parametrically by means of different polynomial orders. Importantly, the corresponding estimates obtained are qualitatively similar.

Finally, Table S6 reports RDD estimates produced using a non-parametric local randomization approach focusing on observations within a small neighbourhood around the cut-off [62]. Columns (2)-(6) show non-parametric RDD estimates based on different choices of bandwidth, around the threshold of 6.5 percent. Results confirm that all findings are consistent, similar in magnitude to our baseline estimates, and still statistically significant.

## Discussion and conclusions

We contribute to the literature by identifying the causal impact of health information on mental health via the effect of a type-2 diabetes diagnosis on clinical depression. We exploit the exogenous cut-off value in the diagnosis of type-2 diabetes provided by a well-established biomarker (glycated haemoglobin) and information on diagnosed clinical depression drawn from rich administrative longitudinal data from Spain by employing a fuzzy regression discontinuity design. In addition, we explore heterogeneity in the effects on mental health by gender, age, bodyweight as well as the role played by weight losses as a potential mechanism leading to changes in the probability of developing clinical depression, following a diabetes diagnosis.

Our results suggest a statically significant impact of health information on diagnosed clinical depression. However, this appears to be driven by gender as well as weight losses eventually occurring after the diabetes diagnosis. More specifically, the overall increase in the risk of developing clinical depression following a diabetes diagnosis appears to be mostly influenced by women. Moreover, the occurrence of weight losses could be one of the possible mechanisms governing the relationship between a diabetes diagnosis and depression. Here differences by gender are also present: whereas a diabetes diagnosis increases the probability of depression among women who did not lose weight, it substantially decreases the risk of clinical depression among men who lost weight. Interestingly, this may suggest a somewhat protective effect of health information via weight losses for male patients with diabetes. In addition, increases in depression appear to be mainly driven by women in the highest category of BMI (> = 30, corresponding to obesity) and are also larger among relatively younger women as well.

In general, the finding that individuals with healthier behavioural patterns present lower levels of mental disorders is also supported by the medical literature [10–16]. As for the different effects by gender, these could be explained on several grounds. For instance, the literature suggests that women might have a higher propensity to clinical depression driven by biological factors [71]. Second, evidence also suggests that depression tend to be underdiagnosed among men [71]. Third, the mental health of individuals, especially that of women, with a high baseline weight may not be significantly affected by weight losses [72]. This might be the case in our data as well, where women present higher rates of obesity as well as a higher average baseline (i.e. before a diabetes diagnosis) BMI if compared to male patients (30.9 vs 29.35, respectively, with the difference being statistically significant as suggested by a pairwise t-test). However, further evidence might be needed to firmly establish the reasons behind heterogeneity in the effects by gender.

Importantly, our results differ from the ones of Gaggero [7], to the best of our knowledge the only paper providing causal evidence on diabetes and mental health, who finds no effects on self-reported mental health in a sample of older individuals. The divergence in results might be due to differences in reliability and precision of key variables including our running variable (glycated haemoglobin coupled with a physician’s diagnosis vs Fasting Plasma Glucose) and outcome variable (diagnosed vs self-reported depression) together with differences in the data employed (as our dataset includes health care records based on actual health care use of a large population of individuals followed over 10 years).

Our results might suggest two possible policy implications. A first recomendation might be that individuals at risk of diabetes should be closely monitored not just to avoid the expected physical health complications associated with T2DM, but also to prevent the onset of other relevant and costly mental health conditions such as clinical depression. In addition, our findings seem to suggest that monitoring should be specifically targeted at women, especially those who did not lose weight following a diagnosis and those who are obese. This may also imply that interventions aimed at reducing psycholigical distress among severely overweight women also include the promotion of healthy behaviours, including following an exercise regimen and a low-carbohydrate diet to decrease bodyweight and this could also help control T2DM. Ultimately, this could be linked to the larger debate around the relevance of changes in health behaviours in influencing mental health, showing that healthier habits such as regular physical activity, healthy eating and adequate sleep can result into improved mental health for people with other chronic conditions beyond T2DM [73–76]. As usual, this study may have some potential limitations. First, as our dataset does not include comprehensive information on the medications prescribed by physicians, we might not be able to account for those pharmaceuticals potentially affecting mental health. Second, the administrative records used here only includes a relatively limited number of variables proxying socioeconomic status. While this might not necessarily be a major issue given that the Spanish health care system is universal and free at the point of delivery, we might not be able to identify potentially informative socioeconomic gradients. Despite such limitations, our study provides novel empirical evidence on the causal impact of health information on mental health, shedding light on gender-based differences in such effects and potential mechanisms through changes in lifestyle behaviours.

## Supplementary Information

**Additional file 1: Table S1.** Summary Statistics - By T2DM Diagnosis Status. **Table S2.** Summary Statistics - By Gender. **Table S3.** Summary Statistics - By Weight Loss Status. **Table S4.** RDD Estimates of the Impact of a T2DM Diagnosis on Depression - Different Cut-offs. **Table S5.** RDD Estimates of the Impact of a T2DM Diagnosis on Depression – Different Polynomials. **Table S6.** Fuzzy RDD Estimates - Non-Parametric Approach.

## Data Availability

The data that support the findings of this study are available from BSA (Badalona Serveis Assistencials) but restrictions apply to the availability of these data, which were used under license for the current study, and so are not publicly available.

## Abbreviation

ADA: American Diabetes Association
COPD: Chronic obstructive pulmonary disease
EU: European Union
FPG: Fasting Plasma Glucose
GADS: Golberg Anxiety and Depression Scale
GP: General practitioner
HRSD: Hamilton Rating Scale for Depression
HbA1c: Glycated hemoglobin
ICPC: International Classification of Primary Care
IEC: International Expert Committee
RDD: Regression discontinuity design
T2DM: Type 2 diabetes mellitus
2SLS: Two-stage least squares

## Footnotes

1 Data from both the Catalan and the Spanish National Statistical Institutes for the year 2010 (the year where the last wave of the data used here was collected) show that sociodemographic characteristics of individuals residing in the city of Badalona seem to match very closely national averages concerning age and gender with small differences in employment/activity (65.5% vs 60.2% in Badalona and Spain, respectively) and share of immigrants (around 15% vs 12.2%). Our sample also appears to closelyresemble main observables of the city of Badalona, with the exception of immigrant status (9.5% vs. 15% in our sample vs the city of Badalona) and employment rate (71% vs. 65.5%). As for all routinely collected administrative datasets, it should be noted that the data used here are drawn from administrative records of all individuals who used health care services in the province of Badalona during the period 2004–2010. As such, these are not meant to be statistically representative of the city of Badalona or the Spanish population and some differences might be expected.

2 These two terms are synonymous and can be used interchangeably.

3 That is, at least 8 over 17 symptoms need to be present to diagnose depression using the Hamilton scale, HRSD; 7 over 18 symptoms using the Golderbg scale, GADS (including at least 5 anxiety symptoms together with 2 depression symptoms); and 20 over 35 symptoms for the 35-item Geriatric scale and at least 5 over 15 for its short-form version, GDRS. Note that although we know that, according to the guidelines of the corresponding local health authority, these three psycometrically validated measures were used by physicians to diagnose clinical depression, our data just include a dummy variable defining a patient’s final diagnosis of clinical depression based on such measures, i.e. our data do not include the specific items/symptoms used within each measure. Yet, a detailed description of each item/symptom can be found [46, 49, 50]. A comprehensive discussion of the differences between depression scales can be found in Edelstein et al. [51].

4 While HbA1c was universally considered the main tool to diagnose diabetes only from 2009 [52], the Spanish health care system (as many other health care systems) was routinely using it to diagnose diabetes during the years covered by this analysis.

5 A series of papers suggest that HbA1c tests administered on previously undiagnosed individuals have generally a very high specificity (i.e., the ability to correctly identify individuals who do not have diabetes) normally around 97% and a more variable sensitivity (i.e., the ability to correctly identify individuals who have diabetes) ranging between around 50% to 83%, depending on the populations considered [54, 55]. Because of the latter, the IEC also recommends that a T2DM diagnosis should be confirmed by a repeated test, unless the diagnosis is clear on clinical grounds [52]. The current practice in Spain [56] is to retest the HbA1c level when the level is close to 6.5% after 3 to 6 months. According to recent evidence, this practice would help identifying more true-positives, potentially raising the sensitivity of the test [57]. Here we employ within-year mean value of HbA1c tests, exploiting the presence of repeated measurements.

6 Note that in our dataset patients usually present multiple HbA1c measurements per year. In order to account for this, our models employ the within-year mean value of HbA1c. Note that results are similar when using the last HbA1c measurement of the corresponding year. In addition, given the differences between type-1 and type-2 diabetes and related treatments, we dropped all individuals with type-1 diabetes.

7 The initial sample includes 123,453 individuals. Since our analysis relies on the information provided by the biomarkers, we include in our sample those individuals with at least one measurement per year, resulting into 13,971 individuals (corresponding to 39,994 obs.). Given that almost all individuals in the sample who are at risk of or have been diagnosed with T2DM present at least one biomarker measurement per year, the exclusion of individuals with no biomarker measurements is unlikely to bias the results of our empirical analysis.

8 The relatively high clinical depression rate might be in part due to the fact that our sample includes older individuals most of whom are diagnosed with T2DM.

9 In this analysis, once individuals are diagnosed with type-2 diabetes, they are also considered diabetics in the subsequent waves. This appears to be a realistic assumption in this case as T2DM is a chronic condition and individuals might eventually achieve what is now defined as “remission” only after drastic lifestyle changes (also note that there still appears to be a debate around the actual definition of a T2DM remission [59].

10 In practice, we allow the function form to vary on either side of the cut-off by including an interaction term between the binary variable $Abov{e}_{it}$ and the normalised running variable.

11 In a recent paper Gelman and Imbens [61] advice against the use of polynomial orders higher than 2, given their poor properties (they appear to be more sensitive to outcome values of far distant observations from the cut-off point and yield inaccurate confidence intervals). Accordingly we use a simple linear parametric approach as our baseline estimates. However, we also present estimates based on polynomials 2–4 as a robustness check.

12 We also produced heteroskedasticity-robust standard errors according to Kolesár and Rothe [64] as these are often recommended when the number of support points around the cut-off is sufficiently large and are based on a smaller bandwidth. Results are similar and available upon request.

13 We also estimated the McCrary test obtaining an estimated log discontinuity in the density of $\widehat{\theta }$= – 0.071 (s.e. 0.0157), failing to reject the null hypothesis of no discontinuity. Similarly, following Cattaneo et al. [68] we obtained a manipulation test score of T =—0.4614 (*p*-value of 0.6445), also suggesting no evidence of systematic manipulation of the running variable.

14 Due to the lack of observations and corresponding limited sample, we could not explore whether the impact of a T2DM on depression might vary by smoking or drinking cessation.

15 We find similar results when considering 65 years-old (these are available upon request).
