# Supplementary material for: Sick and depressed? The causal impact of a diabetes diagnosis on depression
Source: Health Econ Rev. 2023 Jul 3;13:38. doi: 10.1186/s13561-023-00451-w (PMC10316538; doi:10.1186/s13561-023-00451-w)
Supplement: Supplementary file 1 — Additional file 1: Table S1. Summary Statistics - By T2DM Diagnosis Status. Table S2. Summary Statistics - By Gender. Table S3. Summary Statistics - By Weight Loss Status. Table S4. RDD Estimates of the Impact of a T2DM Diagnosis on Depression - Different Cut-offs. Table S5. RDD Estimates of the Impact of a T2DM Diagnosis on Depression – Different Polynomials. Table S6. Fuzzy RDD Estimates - Non-Parametric Approach. [file 13561_2023_451_MOESM1_ESM.docx]

# Additional file 1

Table S1: Summary Statistics - By T2DM Diagnosis Status

|  | (1) | (2) | (3) |
| --- | --- | --- | --- |
|  | Control | Treated | p-value |
| Outcome Variable:  Depression [0,1] | 0.17 | 0.17 | 0.714 |
|  | (0.375) | (0.377) |  |
| T2DM Variables:  Onset of T2DM | 0.00 | 4.86 | 0.000 |
|  | (0.000) | (3.403) |  |
| HbA1C (%) | 6.10 | 6.52 | 0.000 |
|  | (0.425) | (0.505) |  |
| Demographics:  Years of Age | 64.80 | 67.56 | 0.000 |
|  | (12.503) | (10.946) |  |
| Female [0,1] | 0.55 | 0.52 | 0.000 |
|  | (0.498) | (0.500) |  |
| Not Living Alone [0,1] | 0.87 | 0.87 | 0.703 |
|  | (0.335) | (0.333) |  |
| Active [0,1] | 0.33 | 0.19 | 0.000 |
|  | (0.469) | (0.394) |  |
| Immigrant [0,1] | 0.03 | 0.01 | 0.000 |
|  | (0.157) | (0.092) |  |
| Other Conditions:  Hypertension [0,1] | 0.49 | 0.67 | 0.000 |
|  | (0.500) | (0.469) |  |
| Dyslipedimia [0,1] | 0.47 | 0.59 | 0.000 |
|  | (0.499) | (0.492) |  |
| Asthma | 0.04 | 0.05 | 0.698 |
|  | (0.206) | (0.209) |  |
| Neoplasms-cancer [0,1] | 0.06 | 0.07 | 0.000 |
|  | (0.229) | (0.263) |  |
| COPD | 0.04 | 0.06 | 0.000 |
|  | (0.207) | (0.240) |  |
| Observations | 6324 | 16459 | 22783 |

Note: The Table reports summary statistics of the main variables of interest comparing individuals diagnosed with T2DM (treated) and not diagnosed with T2DM (control).

Table S2: Summary Statistics - By Gender

|  | (1) | (2) | (3) |
| --- | --- | --- | --- |
|  | Men | Women | p-value |
| Lifestyle Behaviours:  Body Mass Index | 29.31 | 31.17 | 0.000 |
|  | (4.164) | (5.585) |  |
| Mental Health:  Depression [0,1] | 0.09 | 0.25 | 0.000 |
|  | (0.288) | (0.434) |  |
| Key Variable:  HbA1C (%) | 6.67 | 6.53 | 0.000 |
|  | (1.447) | (1.411) |  |
| Demographics:  Years of Age | 63.90 | 66.19 | 0.000 |
|  | (12.053) | (13.030) |  |
| Not Living Alone [0,1] | 0.91 | 0.85 | 0.000 |
|  | (0.293) | (0.360) |  |
| Active [0,1] | 0.32 | 0.23 | 0.000 |
|  | (0.466) | (0.418) |  |
| Immigrant [0,1] | 0.02 | 0.02 | 0.034 |
|  | (0.123) | (0.133) |  |
| Other Conditions:  Dyslipedimia [0,1] | 0.52 | 0.54 | 0.001 |
|  | (0.500) | (0.498) |  |
| Hypertension [0,1] | 0.53 | 0.64 | 0.000 |
|  | (0.499) | (0.480) |  |
| Observations | 18952 | 20736 | 39688 |

Note: The Table reports summary statistics of the main variables of interest.

Table S3: Summary Statistics - By Weight Loss Status

|  | (1) | (2) | (3) |
| --- | --- | --- | --- |
|  | No Weight Loss | Weight Loss | p-value |
| Lifestyle Behaviours:  Body Mass Index | 30.12 | 30.82 | 0.000 |
|  | (4.851) | (5.042) |  |
| Mental Health:  Depression [0,1] | 0.17 | 0.18 | 0.417 |
|  | (0.376) | (0.380) |  |
| Key Variable:  HbA1C (%) | 6.67 | 6.74 | 0.000 |
|  | (1.329) | (1.258) |  |
| Demographics:  Years of Age | 66.24 | 66.99 | 0.000 |
|  | (10.083) | (10.374) |  |
| Female [0,1] | 0.53 | 0.55 | 0.003 |
|  | (0.499) | (0.497) |  |
| Not Living Alone [0,1] | 0.88 | 0.88 | 0.534 |
|  | (0.327) | (0.331) |  |
| Active [0,1] | 0.19 | 0.18 | 0.063 |
|  | (0.392) | (0.382) |  |
| Immigrant [0,1] | 0.01 | 0.01 | 0.191 |
|  | (0.099) | (0.089) |  |
| Other Conditions:  Dyslipedimia [0,1] | 0.58 | 0.58 | 0.511 |
|  | (0.493) | (0.494) |  |
| Hypertension [0,1] | 0.68 | 0.69 | 0.167 |
|  | (0.467) | (0.463) |  |
| Observations | 7944 | 8244 | 16188 |

Note: The Table reports summary statistics of the main variables of interest.

Table S4: RDD Estimates of the Impact of a T2DM Diagnosis on Depression - Different Cut-offs

|  | (1) | (2) | (3) | (4) | (5) | (6) | (7) | (8) |
| --- | --- | --- | --- | --- | --- | --- | --- | --- |
|  | Cut-off  4.5 | Cut-off 5 | Cut-off  5.5 | Cut-off 6 | Cut-off  6.5 | Cut-off 7 | Cut-off  7.5 | Cut-off 8 |
| T2DM Diagnosis [0,1] | 0.015 | 0.015 | 0.002 | 0.014* | 0.016** | 0.017** | 0.020** | 0.015* |
|  | (0.026) | (0.010) | (0.007) | (0.006) | (0.005) | (0.005) | (0.007) | (0.007) |
| Observations | 34319 | 34319 | 34319 | 34319 | 34319 | 34319 | 34319 | 34319 |

Note: The Table reports RDD estimates on the outcomes of interest, when using a different cut-o s. The alternative cut-o value are 4.5, 5, 5.5, 6, 6.5, 7, 7.5, and 8. Although not shown in the Table, estimates are conditional on a set of covariates, time, areas and GP fixed effects (FE). Robust standard errors are clustered on the running variable. * p < 0.05, ** p < 0.01, *** p < 0.001.

Table S5: RDD Estimates of the Impact of a T2DM Diagnosis on Depression - Different

Polynomials

|  | (1) | (2) | (3) | (4) |
| --- | --- | --- | --- | --- |
|  | Linear | Quadratic | Cubic | Quartic |
| T2DM Diagnosis [0,1] | 0.016** | 0.022*** | 0.024*** | 0.023*** |
|  | (0.005) | (0.005) | (0.006) | (0.006) |
| Observations | 34319 | 34319 | 34319 | 34319 |

Note: The Table reports parametric RDD estimates when using different polynomials of the running variable. Although not shown in the Table, estimates are conditional on a set of covariates, time, areas and GP fixed effects (FE). Robust standard errors are clustered on the running variable. * p < 0.05, ** p < 0.01, *** p < 0.001.

Table S6:Fuzzy RDD Estimates - Non-Parametric Approach

|  | (1) | (2) | (3) | (4) | (5) | (6) |
| --- | --- | --- | --- | --- | --- | --- |
|  | Benchmark | Bandwidth 2 | Bandwidth  1.75 | Bandwidth  1.5 | Bandwidth  1.25 | Bandwidth 1 |
| T2DM Diagnosis [0,1] | 0.016** | 0.008** | 0.008** | 0.007* | 0.009** | 0.011** |
|  | (0.005) | (0.002) | (0.003) | (0.003) | (0.003) | (0.004) |
| Observations | 34319 | 35401 | 33626 | 31793 | 27938 | 24741 |

Note: The Table reports non-parametric RDD estimates of the impact of a T2DM diagnosis on lifestyle behaviours. Each coefficient in the table report the effect of being diagnosed with T2DM on lifestyle behaviours. Although not shown in the Table, estimates are conditional on a set of covariates, time, areas and GP fixed effects (FE). Robust standard errors are clustered on the running variable. * p < 0.05, ** p < 0.01, *** p < 0.001.
